# Expression-profiling of apoptosis induced by ablation of the long ncRNA TRPM2-AS in prostate cancer cell

**DOI:** 10.1016/j.gdata.2014.10.020

**Published:** 2014-11-07

**Authors:** Giovanni Lavorgna, Fulvio Chiacchiera, Alberto Briganti, Francesco Montorsi, Diego Pasini, Andrea Salonia

**Affiliations:** a Division of Oncology/Unit of Urology, URI, IRCCS Ospedale San Raffaele, Milan, Italy; b Department of Experimental Oncology, European Institute of Oncology (IEO), Milan-I, Italy; c Research Doctorate Program in Urology, Magna Graecia University, Catanzaro, Italy

**Keywords:** Prostate cancer, ncRNA, Apoptosis, Microarray, Ion channel

## Abstract

We recently identified the long non-coding RNA (ncRNA) TRPM2-AS as a key regulator of survival in prostate cancer [1]. This essential role, coupled to the TRPM2-AS low-expression levels in healthy tissues, makes this ncRNA a suitable therapeutic target for further clinical studies. To get insights into the survival mechanism controlled by this molecule, we ablated its expression in prostate cancer cells and performed a genome-wide analysis of the transcripts differentially regulated in dying cells. Here, we describe in detail the experimental system, methods and quality control for the generation of the microarray data associated with our recent publication [1]. The data are related to [1]. Data have been deposited to the Gene Expression Omnibus (GEO) database repository with the dataset identifier GSE40687.

**Table:** 

Specifications	
Organism/cell line/tissue	*Homo sapiens*
Sex	Male
Sequencer or array type	Illumina HumanHT-12 V3.0 expression beadchip
Data format	Microarray raw and normalized data: TXT files
Experimental factors	PC3 cells without or with TRPM2-AS expression
Experimental features	Microarray gene expression profiling to identify transcripts that are regulated by TRPM2-AS
Consent	N/A
Sample source location	N/A

## Direct link to the deposited data

Microarray data is deposited at the NCBI Gene Expression Omnibus (GEO) database under GEO Series and is available at the following link: http://www.ncbi.nlm.nih.gov/geo/query/acc.cgi?acc=GSE40687.

## Experimental design, materials and methods

### Cell line

The human, androgen-independent prostate cancer cell line PC3 was obtained from ATCC (CRL-1435, ATCC).

### RNA interference

During an in-silico search for antisense transcripts [2], [3], [4], we have identified TRPM2-AS as antisense transcript in respect to TRPM2 gene, which encodes an oxidative stress-activated ion channel [5]. This long ncRNA is overexpressed in several tumor types [1], [4] and its knock-out leads to massive apoptosis of PC3 and DU145 prostate cancer cells [1]. To gain a molecular understanding of the mechanisms by which TRPM2-AS maintains cell survival in prostate cancer cells, PC3 cells were transfected for 48 h either with a non-specific siRNA or with a siRNA ablating TRPM2-AS RNA.

### Microarray and quality control

**Fig. 1:**
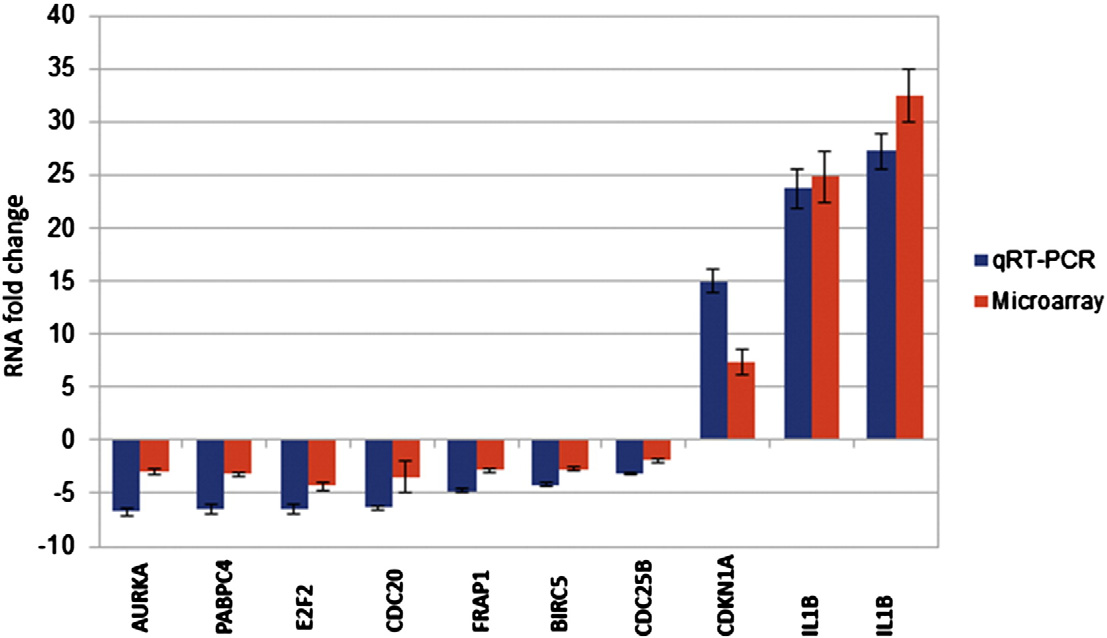
Validation of microarray data by qRT-PCR. Ten transcripts were quantified by qRT-PCR both in control and in TRPM2-AS KO PC3 cells. The resulting expression fold change is plotted against the expression fold change obtained from the Illumina HumanHT-12 V3.0 microarray data. A correlation coefficient of 0.97 was found between the two datasets.

To identify the transcripts that are regulated by TRPM2-AS, we isolated total RNA from four samples (two technical replicates) of PC3 cell lines that had been transfected with the control siRNA or with the experimental siRNA targeting TRPM2-AS expression. The quantity and quality of the RNA samples was measured and assessed by a NanoDrop spectrophotometer and Agilent 2100 Bioanalyzer (Agilent Technologies). Illumina microarray analysis was performed using the Illumina HumanHT-12 V3.0 expression beadchip. Quantitative real-time PCR (qRT-PCR) was used to check the microarray data, analyzing transcript levels that changed in both directions. In particular segments corresponding to the following transcripts were amplified: AURKA, PABPC4, E2F2, CDC20, FRAP1, BIRC5, CDC25B, CDKN1A, IL1B, IL8. Results, shown in Fig. 1, indicated that while, due to the large dynamic range of qRT-PCR [6], microarray data were mostly slightly compressed, there was an excellent qualitative correlation among the two techniques (correlation coefficient: 0.97), thereby validating the microarray experiment.

## Discussion

We report here a dataset composed of microarray gene expression profiling of TRPM2-AS-regulated transcripts. With this experiment, we were able to show that TRPM2-AS coordinates the expression of a large number of genes involved in controlling the survival, the unfolded protein response (UPR) response and the cell-cycle progression in prostate cancer cells. Moreover, targets of existing drugs and treatments were found to be consistently regulated by TRPM2-AS KO, underlying the clinical relevance of this long ncRNA as a novel therapeutic target. According to the dual role of the UPR in promoting cell survival or commitment to programmed cell death [7], several pro-survival molecules and known oncological targets, including the chaperonin HSP90B1 and the oncogenes FYN and AKT1, were also induced by TRPM2-AS KO, suggesting that the dataset as a whole would be particularly valuable for identifying new molecular targets and underlying mechanisms.
